# Amyloid β-Mediated Zn^2+^ Influx into Dentate Granule Cells Transiently Induces a Short-Term Cognitive Deficit

**DOI:** 10.1371/journal.pone.0115923

**Published:** 2014-12-23

**Authors:** Atsushi Takeda, Masatoshi Nakamura, Hiroaki Fujii, Chihiro Uematsu, Tatsuya Minamino, Paul A. Adlard, Ashley I. Bush, Haruna Tamano

**Affiliations:** 1 Department of Neurophysiology, School of Pharmaceutical Sciences, University of Shizuoka, Shizuoka, Japan; 2 Department of Medical Biochemistry, School of Pharmaceutical Sciences, University of Shizuoka, Shizuoka, Japan; 3 Oxidation Biology Unit, The Florey Institute of Neuroscience and Mental Health, The University of Melbourne, Parkville, Victoria, Australia; University of Queensland, Australia

## Abstract

We examined an idea that short-term cognition is transiently affected by a state of confusion in Zn^2+^ transport system due to a local increase in amyloid-β (Aβ) concentration. A single injection of Aβ (25 pmol) into the dentate gyrus affected dentate gyrus long-term potentiation (LTP) 1 h after the injection, but not 4 h after the injection. Simultaneously, 1-h memory of object recognition was affected when the training was performed 1 h after the injection, but not 4 h after the injection. Aβ-mediated impairments of LTP and memory were rescued in the presence of zinc chelators, suggesting that Zn^2+^ is involved in Aβ action. When Aβ was injected into the dentate gyrus, intracellular Zn^2+^ levels were increased only in the injected area in the dentate gyrus, suggesting that Aβ induces the influx of Zn^2+^ into cells in the injected area. When Aβ was added to hippocampal slices, Aβ did not increase intracellular Zn^2+^ levels in the dentate granule cell layer in ACSF without Zn^2+^, but in ACSF containing Zn^2+^. The increase in intracellular Zn^2+^ levels was inhibited in the presence of CaEDTA, an extracellular zinc chelator, but not in the presence of CNQX, an AMPA receptor antagonist. The present study indicates that Aβ-mediated Zn^2+^ influx into dentate granule cells, which may occur without AMPA receptor activation, transiently induces a short-term cognitive deficit. Extracellular Zn^2+^ may play a key role for transiently Aβ-induced cognition deficits.

## Introduction

Memory function normally declines along with aging, and is believed to deteriorate initially because of changes in synaptic function rather than loss of neurons [1]. Some individuals then go on to develop Alzheimer's disease with progressive neurodegeneration [2]–[4]. Neuronal loss is not observed in animal models overexpressing amyloid-β (Aβ) or infused with Aβ, which develop early synaptic alterations but lack the extensive cell death [5], [6]. One of the pathophysiological mechanisms of Aβ is to alter synaptic functions such as long-term potentiation (LTP) and long-term depression (LTD), which are believed to be cellular mechanisms of learning and memory [7], [8]. It has been reported that soluble Aβ oligomers inhibit LTP [7], [9] and facilitate LTD [10]. Thus, the cognitive deficit and memory loss occurring before any prominent neuronal loss, which is observed in patients with mild cognitive impairment (MCI) or early-phase Alzheimer's disease, may be associated with Aβ-induced synapse dysfunctions such as alterations in LTP [11].

Approximately 20–40% of cognitively normal elderly people have evidence of significant brain Aβ-plaque deposition, either from amyloid imaging or CSF Aβ42 concentrations [12]–[14]. Aβ peptides derive from cleavage of the Aβ precursor protein (APP) [15]. APP is initially cleaved by α- or β-secretases, generating large, soluble, secreted fragments (sAPPα and sAPPβ) and membrane-associated carboxy-terminal fragments (CTFs). Aβ peptides of various lengths (e.g., Aβ∼_1_42 and Aβ∼_1_40) are produced after β-secretase cleavage, followed by γ-secretase cleavage. Aβ is normally produced in the brain, where the in vivo concentration in the rodent is estimated to be in the picomolar range [16]. Because Aβ levels in the extracellular fluid are linked to cognitive activity [17], [18], the mechanism of Aβ-mediated modulation in cognition is important to understand cognitive function under both pathological and physiological conditions and also to pursue a strategy to prevent cognitive deficits in the pre-dementia stage of Alzheimer's disease. On the other hand, postmortem studies suggest that the hippocampus and entorhinal cortex are the first brain regions to be affected in Alzheimer's disease [19]–[21].

There is evidence that Zn^2+^ in the extracellular fluid induces amyloid deposition [22], [23], and clinical trials indicate that zinc chelation inhibits Aβ-plaque deposition and improves executive function on a Neuro-psychological Test Battery in patients with Alzheimer's disease [24], [25]. It is likely that amyloid pathology arises in a milieu of constitutively high Zn^2+^ flux. On the other hand, homeostasis of extracellular Zn^2+^ is critical for cognitive activity [26]. Both LTP and object recognition memory are impaired by excess intracellular Zn^2+^ signaling, which is linked to dishomeostasis of extracellular Zn^2+^ induced by excess excitation [27]. It is possible that extracellular Zn^2+^ dynamics is changed in the presence of Aβ and involved in cognitive decline in the pre-dementia stage of Alzheimer disease.

To examine an idea that short-term cognition is transiently affected by a state of confusion in Zn^2+^ transport system due to a local increase in Aβ concentration, a single injection of Aβ∼_1_42 into the dentate gyrus was performed in normal rats, focused on Aβ-mediated Zn^2+^ dynamics.

## Results

### Aβ-induced attenuation of LTP

**Figure 1:**
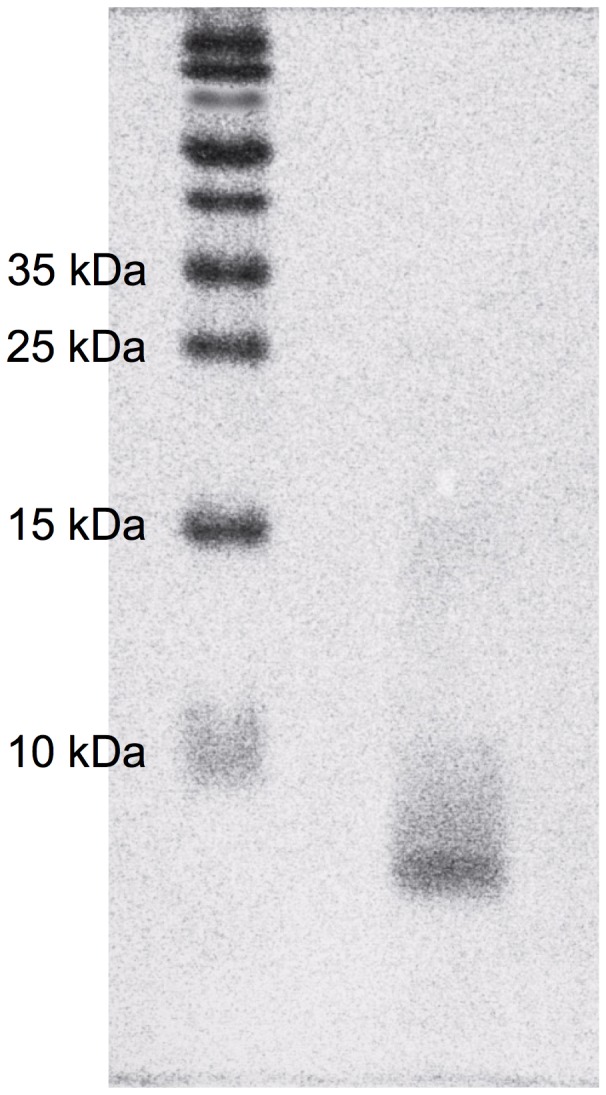
SDS-PAGE of Aβ. Left lane, molecular weigh marker; right lane, sample.

**Figure 2:**
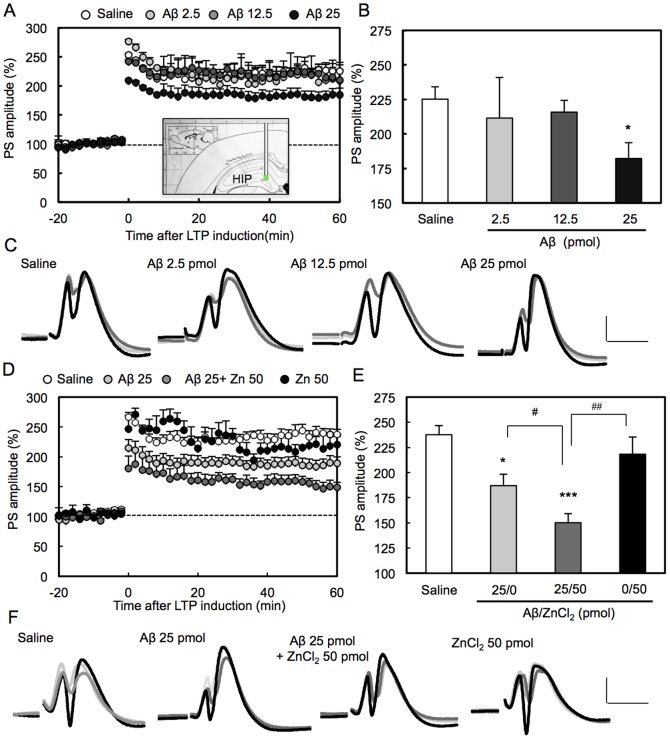
Aβ-induced attenuation of LTP. (A) LTP was induced 1 h after injection of saline (control, n = 7) and Aβ (2.5 (n = 4), 12.5 (n = 4), 25 pmol (n = 9)) in saline (1 µl) via an injection cannula. An inserted picture (coronal section) shows the position of an injection cannula attached to a recording electrode in the hippocampus (HIP). (B) Averaged PS amplitudes for the last 10 min were represented as the magnitude of LTP. *, p<0.05, vs. saline (Dunnett's test). (C) Representative fEPSP recordings at the time −70 min (before injection; light grey line), −20 min (after injection; dark grey line) and 50 min (after tetanic stimulation; black line) are shown. Note that fEPSPs after Aβ injection (dark grey line) were almost the same as those before Aβ injection (light grey line). Scale bar, vertical axis (5 mV), cross axis (10 ms). (D, E and F) LTP was induced 1 h after injection of Aβ (25 pmol, n = 9), Aβ + ZnCl_2_ (50 pmol, n = 9), and ZnCl_2_ (n = 4) in saline. *, p<0.05, ***, p<0.001, vs. saline (n = 7), ^#^, p<0.05, ^##^, p<0.001 (Tukey's test).

**Figure 3:**
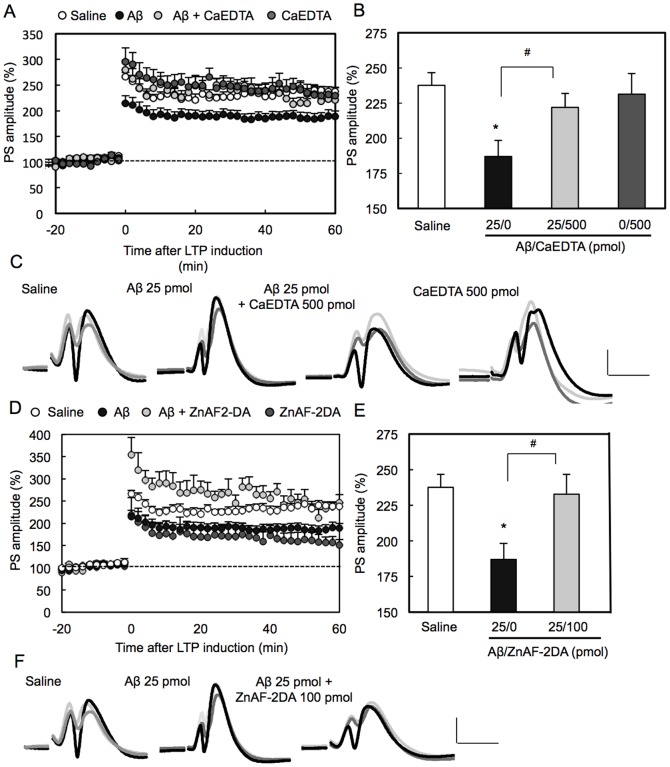
Rescue of Aβ-induced attenuation of LTP with zinc chelators. (A, B, and C) LTP was induced 1 h after injection of Aβ (25 pmol) (n = 9), Aβ + CaEDTA (500 pmol) (n = 8), and CaEDTA (n = 7) in saline (1 µl). *, p<0.01, vs. saline (n = 7), ^#^, p<0.05 (Tukey's test). (D, E, and F) LTP was induced 1 h after injection of Aβ (25 pmol) (n = 9) and Aβ + ZnAF-2DA (100 pmol) (n = 7) in saline (1 µl). *, p<0.05, vs. saline, ^#^, p<0.05 (Tukey's test).

SDS-PAGE showed that Aβ in saline prepared in the present study was mainly monomers. It was estimated that dimers and a very small portion of trimers were also contained in the prepared solution (Fig. 1). LTP was induced 1 h after injection of Aβ into the dentate gyrus via an injection cannula attached to a recording electrode. LTP was significantly attenuated with 25 pmol Aβ (Fig. 2A–2C). The attenuation of LTP was also observed when 25 pmol Aβ was injected into the dentate gyrus 5 min before tetanic stimulation (saline: 265±12% (n = 5), Aβ 194±22% (n = 5), p<0.05 (Dunnett's test) vs. saline). Aβ-induced attenuation of LTP was enhanced by co-injection of 50 pmol ZnCl_2_, which had no effect on LTP (Fig. 2D–2F). On the other hand, Aβ-induced attenuation of LTP was rescued by co-injection of CaEDTA, a membrane-impermeable zinc chelator, and ZnAF-2DA, a membrane-permeable zinc chelator (Fig. 3).

**Figure 4:**
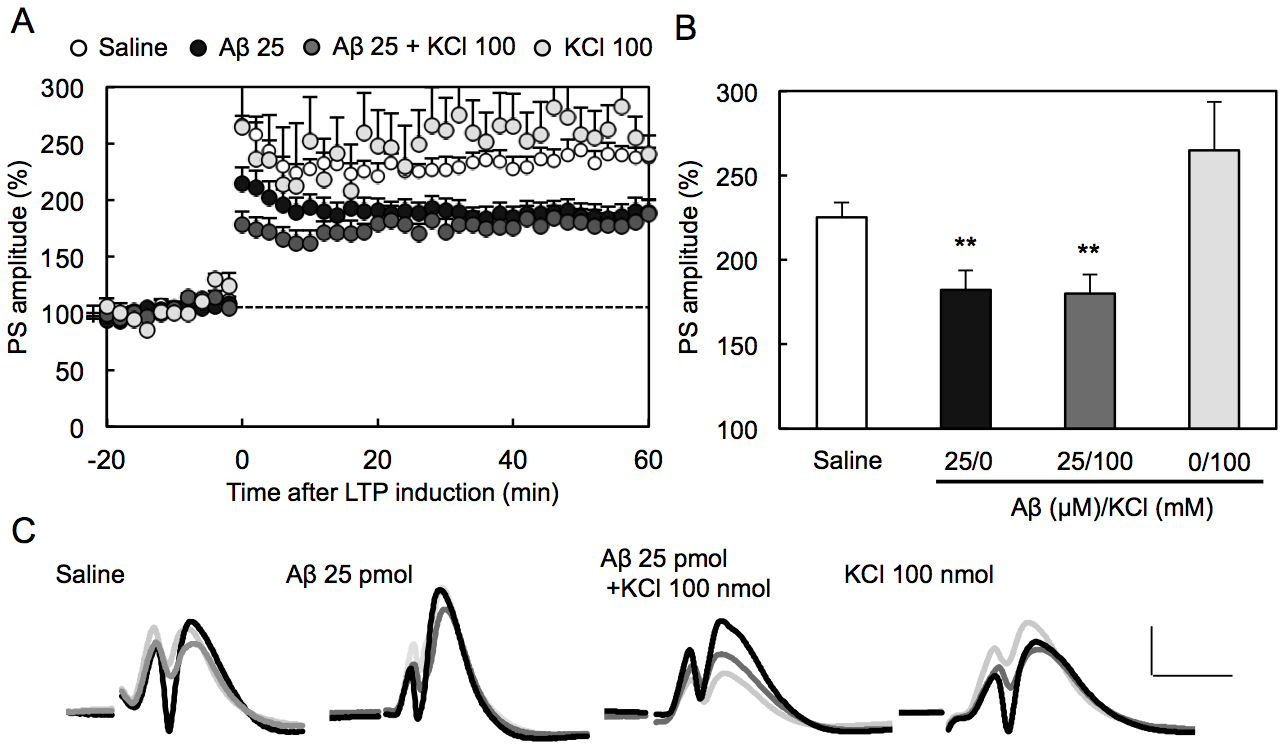
No effect of synaptic excitation on Aβ-induced attenuation of LTP. LTP was induced 1 h after injection of saline (n = 7), Aβ (25 pmol) (n = 9), Aβ + KCl (100 nmol) (n = 7), KCl (n = 4) in saline (1 µl). Note that co-injection of KCl had no significant effect (Dunnett's test) on Aβ-mediated attenuation of LTP. ^**^, p<0.01, vs. saline (Dunnett's test).

The data that the action of Aβ against LTP was rescued with zinc chelators suggest that Zn^2+^ is involved in the action of Aβ. Furthermore, the effect of neuronal depolarization on the action of Aβ was evaluated by using KCl. The recording region where the perforant pathway-dentate granule cell synapses exist was pre-stimulated by co-injection of KCl. However, Aβ-induced attenuation of LTP was not enhanced by co-injection of KCl (Fig. 4), unlike the case of co-injection of ZnCl_2_ (Fig. 2D–2F).

### Aβ-induced memory deficit

**Figure 5:**
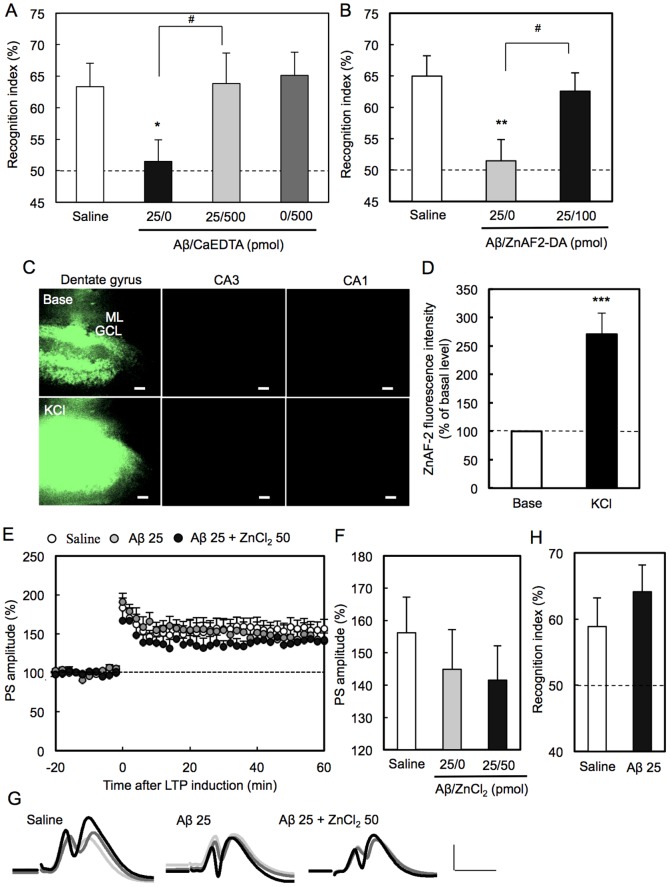
Transiently Aβ-induced memory deficit and its rescue with zinc chelators. (A) The object recognition test was performed 1 h after injection of saline (control) (n = 16), Aβ (25 pmol) (n = 13), Aβ + CaEDTA (500 pmol) (n = 10), and CaEDTA (n = 9) in saline (1 µl). ^*^, p<0.05, vs. saline, ^#^, p<0.05 (Tukey's test). (B) The object recognition test was performed 1 h after injection of saline (control) (n = 16), Aβ (25 pmol) (n = 13), and Aβ + ZnAF2-DA (100 pmol) (n = 12) in saline (1 µl). ^**^, p<0.01, vs. saline, ^#^, p<0.05 (Tukey's test). (C) Brain slices were prepared 1 h after bilateral injection of ZnAF-2DA (100 pmol, 100 µM/1 µl). Intracellular ZnAF-2 fluorescence was measured as the basal level for 30 s and then measured under stimulation with 50 mM KCl for 170 s. Note that intracellular ZnAF-2 fluorescence was observed only in a few slices including the injected area in the dentate gyrus. ML, molecular layer; GCL, granule cell layer. Bars, 50 µm. (D) The data represent the rate (%) of fluorescence intensity in the dentate granule cell layer 170 s after stimulation to that before the stimulation, which was represented as 100% (n = 5). ***, p<0.001 (paired t-test). vs. base. (E and F) LTP was induced 4 h after injection of saline (n = 9), Aβ (25 pmol) (n = 6), and Aβ + ZnCl_2_ (50 pmol) (n = 6) in saline (1 µl). No significant difference, vs. saline (Dunnett's test). (G) Representative fEPSP recordings at the time −250 min (before injection; light grey line), −20 min (after injection; dark grey line) and 50 min (after tetanic stimulation; black line) are shown. Scale bar, vertical axis (5 mV), cross axis (10 ms). (H) The object recognition test was performed 4 h after injection of saline (control) (n = 7) and Aβ (25 pmol) (n = 8) in saline (1 µl). No significant difference, vs. saline (t-test).

To examine whether the attenuation of LTP is linked to an object recognition memory deficit, training of the object recognition test was performed 1 h after injection of Aβ into the dentate gyrus. The exploring time during training was not significantly influenced by injection of Aβ or by co-injection of Aβ and zinc chelators (Dunnett's test). The time exploring objects was 72±4 s (vehicle), 67±3 s (Aβ), 77±9 s (Aβ + CaEDTA), 79±8 s (CaEDTA), and 76±6 s (Aβ + ZnAF-2DA). However, a recognition memory deficit in the test was induced by injection of Aβ, but not by co-injection of CaEDTA or ZnAF-2DA (Fig. 5A and 5B).

ZnAF-2DA has the merits to visualize the area of Zn^2+^ bound to intracellular ZnAF-2, which is produced in cells after ZnAF-2DA injection into the dentate gyrus. To assess the rescue effect of ZnAF-2DA on Aβ-induced impairment of memory, the area containing Zn^2+^ chelated with intracellular ZnAF-2 was checked in brain slices prepared after injection of ZnAF-2DA into the dentate gyrus. Intracellular ZnAF-2 fluorescence was observed in the injected area in the dentate gyrus, but not in the CA3 and CA1 (Fig. 5C). When the brain slices were stimulated with high K^+^, which increases Zn^2+^ influx [27], intracellular ZnAF-2 fluorescence intensity was increased in the injected area (Fig. 5D) and no change in fluorescence intensity was observed in other hippocampal regions, suggesting that intracellular ZnAF-2 in the injected area has a capacity to chelate Zn^2+^ increased by Aβ injection 1 h after ZnAF-2DA injection into the dentate gyrus. The injection of Aβ may induce the influx of extracellular Zn^2+^ in the dentate gyrus, while the co-injection of ZnAF-2DA rescued Aβ-induced impairment of memory (Fig. 5B), probably via blocking Aβ-mediated increase in intracellular Zn^2+^ with intracellular ZnAF-2.

Aβ-induced attenuation of LTP was not observed when LTP was induced 4 h after injection of 25 pmol Aβ into the dentate gyrus (Fig. 5E–5G). When training of the object recognition test was performed 4 h after injection of Aβ into the dentate gyrus, the recognition index was almost the same as the control rats (Fig. 5H).

### Involvement of Aβ-induced Zn^2+^ influx in memory deficit

**Figure 6:**
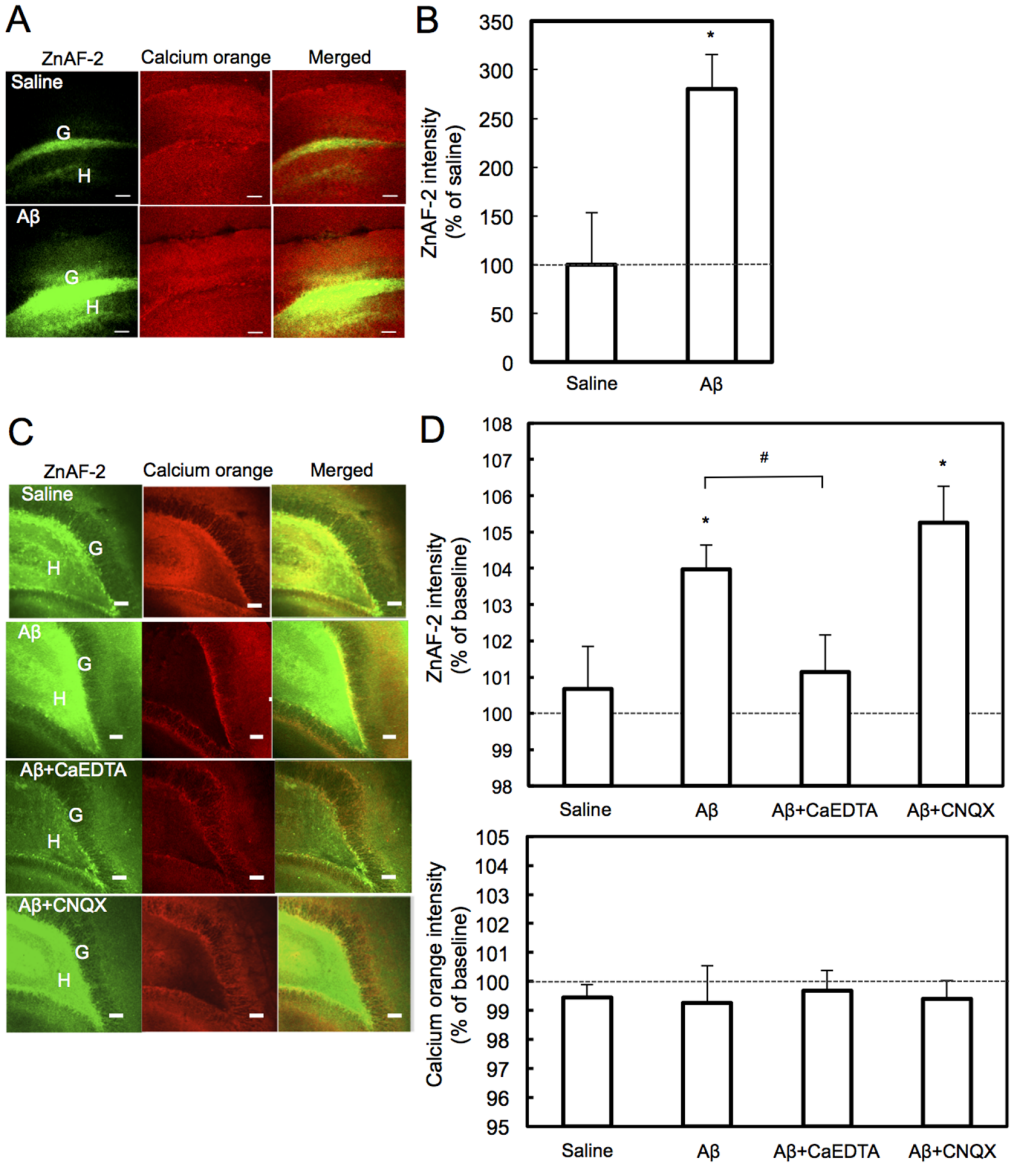
Increase in Aβ-mediated Zn^2+^ influx. (A) Brain slices were prepared 15 min after injection of saline containing ZnAF-2DA (100 pmol) and Aβ (25 pmol) in saline containing ZnAF-2DA (100 pmol) (1 µl) via an injection cannula and stained with calcium orange AM. Note that intracellular ZnAF-2 fluorescence was observed only in a few slices including the injected area in the dentate gyrus and not observed in the CA3 and CA1. G, dentate granule cell layer. H, hilus. Bars, 100 µm. (B) The data represent the ratios (%) of the fluorescence intensity after Aβ injection to that after saline injection that was expressed as 100% (n = 4). ^*^, p<0.05 (t-test). (C) Hippocampal slices doubly stained with ZnAF-2DA and Calcium Orange AM were immersed in 400 µl ACSF containing 1500 nM ZnCl_2_. After measuring each baseline fluorescence for 10 sec, 100 µl of saline (n = 13), 50 µM Aβ in saline (n = 15), 50 µM Aβ +200 µM CNQX in saline (n = 8), and 50 µM Aβ +5 mM CaEDTA in saline (n = 8) was added to hippocampal slices in ACSF and both fluorescence was measured for 160 sec. The images of the dentate gyrus were obtained 160 s after addition. Bars; 50 µm. (D) The data represent the ratios (%) of the fluorescence after the addition (150–160 sec) to the baseline fluorescence (0–10 sec) that was expressed as 100%. ^*^, p<0.05, vs. saline, ^#^, p<0.05 (Tukey's test). No significant difference in calcium orange intensity, vs. saline (Dunnett's test).

Furthermore, Aβ-mediated Zn^2+^ dynamics was checked by co-injection of Aβ and ZnAF-2DA into the dentate gyrus. Aβ increased intracellular Zn^2+^ levels, which were detected with intracellular ZnAF-2, in the injected area 15 min after injection (Fig. 6A and 6B), suggesting that Aβ increases the influx of Zn^2+^ into cells in the injected area. When Aβ was added to hippocampal slices in ACSF, intracellular Zn^2+^ levels in the dentate granule cell layer were not changed (vehicle, 100.16±0.85% (n = 9); Aβ, 99.53±0.91% (n = 9), Dunnett's test). The brain extracellular fluid contains zinc. In hippocampal slices in ACSF containing 1.2 µM ZnCl_2_ at the final concentration, intracellular Zn^2+^ levels were increased in the presence of Aβ (final concentration, 10 µM). This increase was inhibited in the presence of CaEDTA, but not in the presence of 6-cyano-7-nitroquinoxaline-2,3-dione (CNQX), an α-amino-3-hydroxy-5-methyl-4-isoxazolepropionate (AMPA) receptor antagonist (Fig. 6C and 6D). On the other hand, Aβ did not influence intracellular Ca^2+^ levels in the dentate granule cell layer.

## Discussion

The aggregation of Aβ-peptide is widely considered to be the critical step in the pathology of Alzheimer's disease. Small, soluble Aβ oligomers have been shown to be more neurotoxic than large, insoluble aggregates and fibrils [5], [6], [28], [29]. Aβ1-42 is the major component of amyloid deposits in senile plaques [30]. Aβ1-42 promotes amyloid deposition but Aβ1-40 inhibits the process [31]. The soluble forms of Aβ1-42 are the most toxic species that cause neuronal damage in the brains of Alzheimer's disease patients [32]. Zinc plays an important role in Aβ aggregation process [33]–[35]. It is possible that the interaction between Aβ and Zn^2+^ in the extracellular compartment is involved in cognitive deficits in the pre-symptomatic phase of Alzheimer's disease.

In the dentate gyrus of the hippocampus, each granule cell receives two distinct inputs from the entorhinal cortex known as the medial and lateral perforant pathways (MPP and LPP, respectively). MPP fibers originate from the medial entorhinal area and terminate in the middle one-third of the molecular layer of the hippocampus where minimally stained by Timm's sulfide-silver method to detect zinc in the presynaptic vesicles [36]–[38]. Electrical stimulation of MPP fibers produces a characteristic waveform of evoked field excitatory postsynaptic potential [fEPSP] superimposed by a population spike (PS) [39]–[41]. Judging from the PS amplitude recorded in the present study, it is estimated that LTP was induced by tetanic stimulation to the MPP [41], which is non-zincergic [42]. Thus it is estimated that extracellular Zn^2+^ concentration is hardly increased during MPP LTP (dentate gyrus LTP) induction. In the present study, the action of Aβ in LTP and cognitive activity was examined under the basal (static) condition. LTP was attenuated by local injection of Aβ into the recording region 5 min or 1 h before tetanic stimulation. Aβ-induced attenuation of LTP was enhanced by co-injection of ZnCl_2_. It is possible that the basal levels of extracellular Zn^2+^ are involved in the action of Aβ.

The basal concentration of extracellular Zn^2+^ is estimated to be around 10 nM [43] and higher than that (<1 nM) of intracellular (cytosolic) Zn^2+^ [44], [45]. An idea that Aβ-mediated Zn^2+^ influx into dentate granule cells affects LTP was examined by co-injection of zinc chelators. The attenuation of LTP was rescued with CaEDTA to block the influx of extracellular Zn^2+^ [46] and, the rescue effect was also observed by co-injection of ZnAF-2DA, which blocks intracellular Zn^2+^ signaling [47], supporting the idea that Aβ-mediated Zn^2+^ influx affects LTP.

If LTP is involved in learning of object recognition, it is estimated that Aβ simultaneously affects cognitive activity during the learning. Aβ transiently induced the impairment of memory in agreement with the attenuation of LTP. Furthermore, Aβ-induced impairment of memory was rescued with both CaEDTA and ZnAF-2DA. The present study indicates that cognitive activity is transiently affected via Aβ-induced attenuation of LTP and that a state of confusion in Zn^2+^ transport system in the dentate gyrus may be involved in the Aβ-induced cognition deficit.

To examine whether neuronal depolarization aggravates Aβ action, Aβ was co-injected with KCl. KCl shortly induces depolarization of all neurons in the injected area prior to LTP induction. KCl had no effect on Aβ-induced attenuation of LTP. It is likely that extracellular Zn^2+^ is minimally increased in the recording area after injection of KCl into the dentate gyrus because of no release of Zn^2+^ from the MPP, resulting in no enhancement of Aβ-induced attenuation of LTP. In contrast, it is possible that Aβ-mediated attenuation of LTP is enhanced after neurons are depolarized in the presence of Aβ at zincergic synapses because of the increase in extracellular Zn^2+^.

Cleary et al. [28] report that soluble oligomeric forms of Aβ, including trimers and dimers, are both necessary and sufficient to disrupt cognitive activity in a manner that is rapid, potent and transient; they impair cognitive function without permanent neurological deficits. Furthermore, there are some studies on memory deficits after a single injection of Aβ into the brain; Lesné et al. [5] show that a 56 kDa form of Aβ purified from Tg2576 mouse brain disrupts maze performance when injected into the lateral ventricle of rats. Soluble Aβ dimers, isolated from cerebral cortex of AD subjects, inhibit LTP, reduce dendritic spine density in rodent hippocampus, and disrupt memory in normal rats [6]. While multiple forms of Aβ impair cognitive activity, there are significant differences in effective concentration and potency [48].

On the other hand, Aβ has been shown to disturb intracellular calcium homeostasis that may alter calcium-related signal transduction pathways [49]. Intracellular Ca^2+^ levels are increased in astrocytes exposed to Aβ [50]. Aβ increases intracellular Ca^2+^ concentration by Ca^2+^ influx through calcium-permeable channels formed by Aβ and those channels are Zn^2+^-sensitive at high doses (50∼1000 µM) [51], [52]. Zn^2+^ passes through calcium-permeable channels such as calcium-permeable AMPA receptors and N-methyl-D-aspartate (NMDA) receptors and high doses of Zn^2+^ block NMDA receptors and voltage-dependent calcium channels [46], [53], [54]. Multiple forms of Aβ are produced in the extracellular compartment and changed by the surrounding environment such as the presence of heavy metals. The changes may be markedly dynamic in the brain. Aβ-mediated Zn^2+^ dynamics was assessed after Aβ injection into the dentate gyrus of rats. Intracellular Zn^2+^ levels were increased only in the injected area. Furthermore, in vitro hippocampal slice experiments indicated that Aβ also increased intracellular Zn^2+^ levels in the dentate granule cell layer in ACSF containing 1.2 µM ZnC1_2_ but not in ACSF without Zn^2+^. Aβ-mediated Zn^2+^ influx, which did not influence intracellular Ca^2+^ levels, was inhibited in the presence of CaEDTA, but not in the presence of CNQX, which blocks glutamatergic neuron activation. These data suggest that extracellular Zn^2+^ is critical for Aβ-mediated Zn^2+^ influx into dentate granule cells. Aβ-mediated Zn^2+^ influx may occur under the basal condition that is independent on AMPA receptor activation. It is possible that Zn^2+^ influx into dentate granule cells occurs through zinc-permeable channels (ionophores) formed by Aβ and the channels interact with multiple receptors such as NMDA receptors and prion protein [55]. Thus Zn^2+^ potentially flows into the perforant pathway including MPP terminals in the same manner. Cognitive activity is transiently affected by unknown mechanism after intracellular Zn^2+^ levels are excessively increased by Aβ-mediated Zn^2+^ influx. Aβ-induced attenuation of MPP LTP, which is induced NMDA receptor-dependently [22], [41], may be involved in cognitive deficits. This type of LTP is sensitive to excess Zn^2+^ [56]. It is necessary to identify target molecules of excess Zn^2+^ to understand the mechanism of Aβ-induced attenuation of MPP LTP.

In conclusion, the present study indicates that Aβ-mediated Zn^2+^ influx into dentate granule cells, which may occur without AMPA receptor activation, transiently induces a short-term cognitive deficit. Extracellular Zn^2+^ may play a key role for transiently Aβ-induced cognition deficits. In subjects in the pre-dementia stage of Alzheimer's disease, their cognitive deficits are limited to memory alone and/or their everyday abilities are preserved. A novel strategy focusing on a state of confusion in Zn^2+^ transport system due to Aβ may contribute to not only understanding transient cognition deficits in the pre-dementia stage of Alzheimer's disease but also preventing pathogenesis of Alzheimer's disease.

## Materials and Methods

### Animals and chemicals

Male Wistar rats (6–8 weeks of age, Japan SLC (Hamamatsu, Japan) were used for the experiments. They were housed under the standard laboratory conditions (23±1°C, 55±5% humidity) and had access to tap water and food ad libitum. All experiments were performed in accordance with the Guidelines for the Care and Use of Laboratory Animals of University of Shizuoka that refer to American Association for Laboratory Animals Science and the guidelines laid down by the NIH (NIH Guide for the Care and Use of Laboratory Animals) in the USA. The Animal Experiment Committee of University of Shizuoka approved all protocols for animal experiments (#136043).

Synthetic Aβ1-42 for human was purchased from ChinaPeptides (Shanghai, China). Aβ was dissolved in saline and immediately used when the experiments were performed. ZnAF-2DA (K_d_ = 2.7×10^−9^ M for zinc), a membrane-permeable zinc indicator, was kindly supplied from Sekisui Medical Co., LTD (Tokai, Japan). ZnAF-2DA is taken up into the cells through the cell membrane and is hydrolyzed by esterase in the cytosol to yield ZnAF-2, which cannot permeate the cell membrane [57], [58]. Calcium orange AM, a membrane-permeable calcium indicator, was purchased from Molecular Probes, Inc. (Eugene, OR). These indicators were dissolved in dimethyl sulfoxide (DMSO) and then diluted to artificial cerebrospinal fluid (ACSF) containing 119 mM NaCl, 2.5 mM KCl, 1.3 mM MgSO_4_, 1.0 mM NaH_2_PO_4_, 2.5 mM CaCl_2_, 26.2 mM NaHCO_3_, and 11 mM D-glucose (pH 7.3).

### Sodium dodecyl sulfate-polyacrylamide gel electrophoresis

Sodium dodecyl sulfate-polyacrylamide gel electrophoresis (SDS-PAGE) was performed according to Laemmli's method [59]. Aβ (50 µM, 22.5 µl) in saline was added to a sample buffer (7.5 µl) (final concentration, 37.5 µM). One hour later, the sample was loaded (30 µl/lane) onto 4% (w/v) acrylamide stacking gel (125 mM Tris-HCl (pH 6.8), 0.1% (w/v) SDS, 0.05% ammonium peroxodisulfate (APS), 0.05% N,N,N',N'-tetramethylethylenediamine )TEMED)) and 18% )w/v) acrylamide running gel (375 mM Tris-HCl )pH 8.8), 0.1% (w/v) SDS, 0.05% APS, 0.05% TEMED). After electrophoresis, Aβ was detected with CBB (Coomassie Brilliant Blue) staining.

### Slice preparation and imaging

Rats were anesthetized with ether and decapitated. The brain was quickly removed and immersed in ice-cold choline-ACSF containing 124 mM choline chloride, 2.5 mM KCl, 2.5 mM MgCl_2_, 1.25 mM NaH_2_PO_4_, 0.5 mM CaCl_2_, 26 mM NaHCO_3_, and 10 mM glucose (pH 7.3) to suppress excessive neuronal excitation. Transverse hippocampal slices (400 µm) or coronal brain slices (400 µm) were prepared using a vibratome ZERO-1 (Dosaka, Kyoto, Japan) in an ice-cold choline-ACSF. Slices were then maintained in ACSF at 25°C (for at least 1 h). All solutions used in the experiments were continuously bubbled with 95% O_2_ and 5% CO_2_.

For intracellular zinc and calcium imaging, the hippocampal slices were loaded with 10 µM ZnAF-2DA and 10 µM calcium orange AM, respectively, for 30 min and then transferred a chamber filled with ACSF to wash out extracellular ZnAF-2DA and calcium orange AM for at least 30 min. The hippocampal slices were transferred to a recording chamber filled with ACSF. The fluorescence of ZnAF-2 (excitation, 488 nm; monitoring, 505–530 nm) and calcium orange (excitation, 543 nm; monitoring, above 560 nm) was measured in the granule cell layer of the dentate gyrus with a confocal laser-scanning microscopic system LSM 510 (Carl Zeiss), equipped with the inverted microscope (Axiovert 200 M, Carl Zeiss), at the rate of 1 Hz through a 10× objective.

To identify the area containing Zn^2+^ chelated with intracellular ZnAF-2, coronal brain slices were prepared 15 min or 1 h after injection of 1 µl ZnAF-2DA (100 µM) or 1 µl ZnAF-2DA (100 µM) containing Aβ (25 µM) into the dentate granule cell layer of unanesthetized rats at the rate of 0.25 µl/min for 4 min through injection cannulae. The fluorescence of ZnAF-2 was measured in the dentate granule cell layer in the same manner. In another experiment, brain slices were loaded with 10 µM calcium orange AM for 30 min and the fluorescence of ZnAF-2 and calcium orange was measured in the dentate granule cell layer in the same manner.

### *In vivo* Dentate Gyrus LTP

We used the surgical procedure described previously with a slight modification [41]. Male rats were anesthetized with chloral hydrate (400 mg/kg) and placed in a stereotaxic apparatus. A bipolar stimulating electrode and a monopolar recording electrode made of tungsten wire attached to an injection cannula (internal diameter, 0.15 mm; outer diameter, 0.35 mm) were positioned stereotaxically so as to selectively stimulate the perforant pathway while recording in the dentate gyrus. The electrode stimulating the perforant pathway was positioned 8.0 mm posterior to the bregma, 4.5 mm lateral, 3.0–3.5 mm inferior to the dura. A recording electrode was implanted ipsilaterally 4.0 mm posterior to the bregma, 2.3–2.5 mm lateral and 3.0–3.5 mm inferior to the dura. All the stimuli were biphasic square wave pulses (200 µs width) and their intensities were set at the current that evoked 40% of the maximum PS amplitude. Test stimuli (0.05 Hz) were delivered at 20 s intervals to monitor PS amplitude.

At the beginning of the experiments, input/output curves were generated by systematic variation of the stimulus current (0.1–1.0 mA) to evaluate synaptic potency. After stable baseline recording for at least 30 min, agents in 1 µl saline were locally injected into the dentate gyrus of anesthetized rats at the rate of 0.25 µl/min for 4 min via an injection cannula attached to a recording electrode. LTP was induced by delivery of high-frequency stimulation (HFS; 10 trains of 20 pulses at 200 Hz separated by 1 s) 1 h or 4 h after injection and recorded for 60 min. PS amplitudes (test frequency: 0.05 Hz) were averaged over 120-second intervals and expressed as percentages of the mean PS amplitude measured during the 15-min baseline period perfused with ACSF prior to the injection, which was expressed as 100%. PS amplitudes for the last 10 min were also averaged and represented as the magnitude of LTP. In all experiments, we confirmed the location of the recording/injection sites after completing the experiments. The stimulation artifact was removed at the gaps in the representative traces.

### Object recognition memory

LTP recording and the object recognition test were separately done. Rats were placed for 10 min into an open field, which was a 70×60 cm arena surrounded by 70 cm high walls, made of a black-colored plastic. Twenty-four hours after open field exploration, agents in saline (1 µl) were bilaterally injected via injection cannulae into the dentate gyrus of unanesthetized rats at the rate of 0.25 µl/min for 4 min. One hour or four hours after injection, rats were trained and tested in a novel object recognition task. Training in the object recognition task took place in the same area used for the open field exploration. The open field exploration was thus used as a context habituation trial for the recognition memory task. The object recognition test requires that the rats recall which of two earthenware objects they had been previously familiarized with. Training was conducted by placing individual rats into the field, in which two identical objects (objects A1 and A2; sake bottle) were positioned in two adjacent corners, 15 cm from the walls. Rats were left to explore the objects for 5 min. Rats were not used for the test when the total of the object exploration time was less than 20 s. In the test given 1 h after training, the rats explored the open field for 3 min in the presence of one familiar (A) and one novel (B; cup) object. Behavior of rats was recorded with a video camera during the training and the test, and then two persons independently measured exploratory time and the averaged time was used. All objects presented similar textures, colors and sizes, but distinctive shapes. A recognition index calculated for each rat was expressed by the ratio T_B_/(T_A_+T_B_) [T_A_ =  time spent to explore the familiar object A; T_B_ =  time spent to explore the novel object B]. Between trials the objects were washed with 70% ethanol solution. Exploration was defined as sniffing or touching the object with the nose and/or forepaws. We confirmed that there was no preference for the objects used.

### Data analysis

For statistical analysis, Student's paired *t*-test was used for comparison of the means of paired data. For multiple comparisons, differences between treatments were assessed by one-way ANOVA followed by post hoc testing using the Dunnett's test or the Tukey's test (the statistical software, GraphPad Prism 5). A value of p<0.05 was considered significant. The Dunnett's test was used to compare between the control and treatments. The Tukey's test was used to compare between treatments in addition to the comparison between the control and treatments. Data were expressed as means ± standard error. The results of statistical analysis are described in each figure legend.
